# Translation calibration of inverse-kappa goniometers in macromolecular crystallography

**DOI:** 10.1107/S0108767311004831

**Published:** 2011-03-15

**Authors:** Sandor Brockhauser, Kristopher I. White, Andrew A. McCarthy, Raimond B. G. Ravelli

**Affiliations:** a European Molecular Biology Laboratory, 6 rue Jules Horowitz, BP 181, 38042, Grenoble, France; b Unit of Virus Host–Cell Interactions, UJF–EMBL–CNRS, UMI 3265, 6 rue Jules Horowitz, 38042, Grenoble, Cedex 9, France

**Keywords:** kappa goniometer, macromolecular crystallography, reorientation, calibration

## Abstract

Precise and convenient crystal reorientation is of experimental importance in macromolecular crystallography (MX). The development of multi-axis goniometers, such as the ESRF/EMBL mini-κ, necessitates the corresponding development of calibration procedures that can be used for the setup, maintenance and troubleshooting of such devices. While traditional multi-axis goniometers require all rotation axes to intersect the unique point of the sample position, recently developed miniaturized instruments for sample reorientation in MX are not as restricted. However, the samples must always be re-centred following a change in orientation. To overcome this inconvenience and allow the use of multi-axis goniometers without the fundamental restriction of having all axes intersecting in the same point, an automatic translation correction protocol has been developed for such instruments. It requires precise information about the direction and location of the rotation axes. To measure and supply this information, a general, easy-to-perform translation calibration (TC) procedure has also been developed. The TC procedure is routinely performed on most MX beamlines at the ESRF and some results are presented for reference.

## Abstract 2

A rapid, easy-to-perform translation calibration procedure has been developed for use with the EMBL/ESRF mini-κ goniometer head and for other inverse-kappa goniometers designed for macromolecular crystallography. Regular calibration ensures the precision of experiments that rely on many degrees of freedom in crystal reorientation.

## 1. Introduction

Multi-axis goniometers have long been common in both the realm of small-molecule crystallography as well as in the early years of macromolecular crystallography (MX), as summarized by Helliwell (1992 ▶). Early devices were limited by the risk of potential collisions with other beamline elements, resulting in their replacement by single-axis goniometer setups on modern beamlines. A renewed focus on miniaturization and collision prevention has led to the development of devices that integrate seamlessly with many positioning systems designed for MX (McCarthy *et al.*, 2009 ▶; Wang *et al.*, 2008 ▶; Jain & Stojanoff, 2007 ▶; Shi *et al.*, 2006 ▶; Skinner & Sweet, 1998 ▶). As a result, the use of multi-axis goniometry in MX has risen steadily in recent years. By greatly expanding the range over which a given sample can be reoriented, multi-axis goniometer systems provide additional freedom in the design of diffraction experiments. More importantly, they solve a number of orientation-related problems that are often insurmountable on single-axis setups (Bricogne *et al.*, 2005 ▶).

One such problem in MX is radiation damage (Blake & Philips, 1962 ▶), which has proven to be a major driving factor behind a great deal of recent innovations in MX because of its role in undermining MAD (multiple-wavelength anomalous dispersion) experiments (Hendrickson, 1991 ▶). Aligning an even-fold (2×, 4×, 6×) symmetry axis along the spindle allows one to record Bijvoet pairs (a reflection and the Friedel pair of its symmetry equivalent, *e.g.* 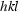 and 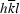) on the same diffraction image. This will not overcome radiation damage; however, it minimizes the radiation-damage-induced non-isomorphism within these Bijvoet pairs, resulting in more accurate anomalous differences. This method can also be performed on single-axis goniometers or non-automated multi-axis instruments, but limitations in available rotational degrees of freedom limit its usefulness. This is due to the difficulties in properly aligning the twofold symmetry axis of a sample along the spindle axis (Dauter, 1999 ▶).

Dauter also discusses the most common problems associated with complex structures containing large unit-cell axes, leading to reflection overlaps that occur when using an oscillation method. In such cases, aligning the densest reciprocal-space vector (commonly corresponding to the longest unit-cell axis) along the spindle is often advantageous. Note that the cell alignment can result in a blind zone (Dauter, 1999 ▶), which does not allow for the collection of a full data set in such an orientation. Finding a slightly tilted alignment where the full data set can be collected while the long axis approaches the spindle as much as possible is a good compromise, but such a precise realignment is only possible in many cases with a multi-axis system. A less elegant but commonly used alternative is to manually bend the pin on which the sample is mounted, but such an approach is risky and not optimal or amenable for automation.

The greater rotational freedom afforded by multi-axis systems is useful with respect to phasing strategies as well. Substantial dichroism and anisotropy in resonant scattering in X-ray data collected from selenated proteins near the Se *K* edge have been noted by Bricogne *et al.* (2005 ▶). They have subsequently proposed a methodology for optimizing the anomalous phasing signal obtained from single- or multiple-wavelength anomalous diffraction (SAD, MAD) experiments based on a crystal alignment relative to the incident-beam polarization and the spindle orientation (Schiltz & Bricogne, 2008 ▶, 2009 ▶; Joosten *et al.*, 2009 ▶).

These studies, as well as reference-beam diffraction measurements (Pringle & Shen, 2003 ▶; Shen, 2003 ▶), present compelling arguments for the use of multi-axis goniometers, which all rely on an accurate and precise knowledge of the direction vectors of the goniometer rotational axes.

**Figure 1:**
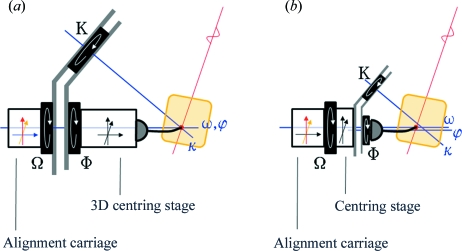
In the case of a traditional κ system (*a*), the alignment carriage moves the perfectly adjusted goniometer along different directions to meet the beam (red), the focal plane of the centring view (orange) by the ϕ and ω axes, and the centring point by the κ axis (blue). The three-dimensional centring stage is then used to move the sample to the centring point. In the case of an inverse-κ system (*b*), the centring stage is placed between the ω and κ axes, allowing for miniaturization of the instrument and reducing the risk of collisions during operation. Since the blue direction of the alignment carriage for adjusting the κ axis is no longer used, re-purposing it for sample centring reduces the scope of the centring stage to two dimensions, allowing a further miniaturization as implemented in the ESRF/EMBL mini-κ system.

The κ goniometer is one of the most common types of multi-axis goniometer used in diffraction studies today. In the case of such an instrument, the axes rotating the sample carry one another. For the three-axis case, they are called ω, κ and ϕ, where ω carries κ, which in turn carries ϕ (Paciorek *et al.*, 1999 ▶). Traditional κ-axis goniometer systems describe a centring alignment system on the ϕ axis and assume that all the rotation axes intersect a unique point along the X-ray beam (see Fig. 1 ▶ *a*). When using the centring alignment system, any given sample can be centred at an arbitrary datum (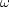, 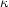, 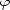) by moving it to this unique point. This setup thus guarantees that, if all hardware is properly aligned, the sample will remain centred during any subsequent rotation. In the case of an inaccurate system in which the axes do not intersect at a unique point, the centring must be done about the axis of data collection, which should also be aligned to intersect the beam at that point of centring. Although multiple-axis data collection is not supported by such a setup, each axis can be scanned one at a time following realignment and re-centring. Miniaturized true-kappa-geometry goniometers are available at the Structural Biology Center at the APS (Rosenbaum & Westbrook, 1997 ▶) and at the Australian Light Source.

**Figure 2:**
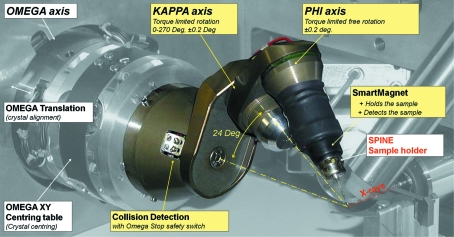
EMBL/ESRF mini-κ goniometer head (MK3) following the inverse-κ design as mounted on the MD2M goniometer of the MX beamline ID14-4 at the ESRF.

Inverse-κ systems (Fig. 1 ▶ *b*) address some of the potential problems with multi-axis systems by moving the centring device out from behind the final rotation axis and in front of the reorientation axes (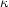, 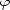). The ESRF/EMBL-developed mini-κ goniometer head (MK3) is one such system (Fig. 2 ▶).^1^ It has been specifically designed for use with 22 mm European SPINE standard cryopins (Cipriani *et al.*, 2006 ▶). On spatially limited MX beamlines, the MK3 provides a good balance between reorientation possibilities and collision avoidance. When mounted on a single-axis host goniometer, the mini-κ head offers most of the functionality of a traditional κ or three-circle goniometer without the risk of collision inherent to such devices. The MK3 is composed of a motorized ϕ shaft mounted on a motorized κ arm, which in turn is mounted on the ω axis or data-collection spindle of a single-axis goniometer designed for MX with centring and alignment features. Rotation about the κ axis is limited to the range [0°, 260°]. When mounted on a diffractometer such as the MD2 (MAATEL, Voreppe, France) (Perrakis *et al.*, 1999 ▶), the angle between the ω and κ axes is 24°, and the ω axis is parallel to the ϕ axis. Similar inverse-κ devices have also been developed for MX beamlines (Shi *et al.*, 2006 ▶; Glettig *et al.*, 2009 ▶).

The use of multi-axis goniometers for high-accuracy reorientation is only reliable if all of the direction and location vectors of the rotation axes are precisely known. The assessment of the misalignment in the direction vectors and subsequent calibration has been addressed by Paciorek *et al.* (1999 ▶). In the case of inverse-κ-axis goniometers, an additional level of complexity must also be addressed in order to define the location vectors. When centring a sample onto the ω axis, the reorientation axes (κ and ϕ) are also translated and will not meet at the centring point. As a direct consequence, every reorientation must be followed by a translational re-centring to keep the sample in the axis of the beam. This design does not offer a unique point of intersection for all of the rotation axes, and the multi-axis goniometer head must be supported by a reliable translation stage as a result. One’s ability to perform sample positioning is thus limited by the reliability, accuracy and precise configuration of the motors responsible for translational motion.

A variety of different industrial solutions have been developed for calibrating and adjusting goniometer systems. The choice of method is determined by the precision and accuracy required for the experiments. Interferometry- (*e.g.* LP30 from FEANOR, Tallinn, Estonia), capacitance- (*e.g.* Elite CPL290 from LION Precison, St Paul, MN, USA) and microscopy-based tools have all been implemented for different systems based on such constraints, and each requires a dedicated instrumentation setup. Here we present a microscopy-based method that takes advantage of standard MX instrumentation and already-implemented projection-based algorithms [*i.e.* automatic or three-click centring for single-axis goniometer heads (Lavault *et al.*, 2006 ▶; Jain & Stojanoff, 2007 ▶)] to allow for rapid calibration of inverse-κ goniometer systems such as the MK3. This method balances the general requirement in MX for spatial resolution of the order of several microns with the practical need for a repeatable calibration technique that is easily performed, either manually or automatically, on current hardware in a matter of minutes. While more rigorous calibration is certainly necessary on occasion, for example during goniometer commissioning, the time required to perform such procedures interferes with beamline administration and such methods are thus only feasible during downtime. The calibration procedure presented here is flexible and can be scaled up to meet such demands.

The current scheme for calibrating the mini-κ goniometer system relies on a translation calibration (TC) method that allows for the determination of the direction and location of the rotation axes required to maintain the sample position over the course of a reorientation. This paper addresses the TC method, outlines a specific technique developed for use on the beamlines, and provides practical suggestions for ways to ensure that a proper calibration has been performed. It also discusses how to diagnose potential systematic problems in the components of the sample-positioning system responsible for such translational movements.

## 2. Methodology

TC of the inverse-κ system provides a description of the direction vector 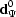 and location vector 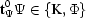 of the κ- and ϕ-rotation axes in a zero-valued goniometer setting. The location vector is defined in the space of the motorized translation-stage axes 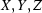 and provides the motor positions needed to move a given point-like object to an arbitrarily defined reference point in real space. By choosing the reference point on the ω axis, the translation-space coordinates become independent of the actual ω position. Furthermore, in the special case when the reference point is chosen as the intersection of the ω axis and the beam where the sample is normally placed during the diffraction experiment, then moving the point to this reference simply means centring the given point. As such, the translation-space coordinates of a given point can be measured by retrieving the translation motor positions after centring the point in question. Note that although the point occupied by a mounted sample is rotating in real space when the ω axis is turned, its place in translation space does not change because the translation stage stays at the same settings and rotates together with the point. When κ- and ϕ-rotation settings are non-zero and a 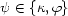 rotation is applied on any of those axes, the location of the sample in the translation space changes as well.

### 2.1. Translation correction

**Figure 3:**
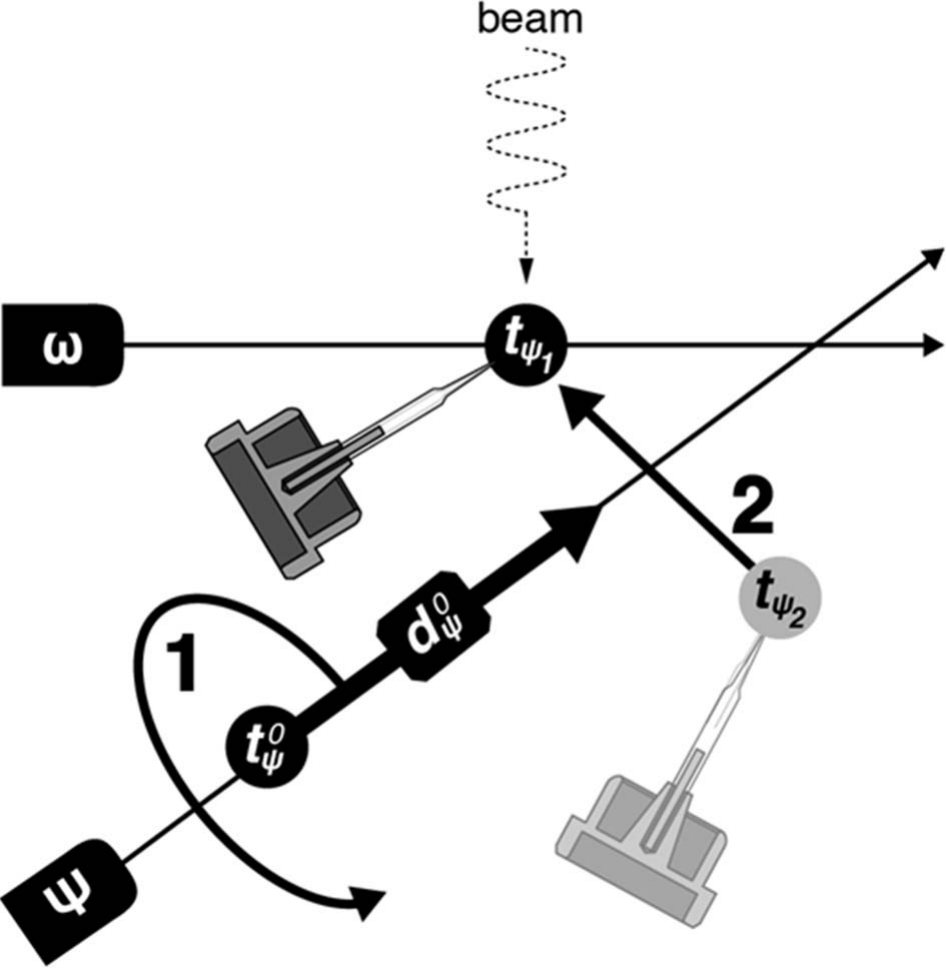
Reorientation of a sample about the κ or ϕ axis of an inverse-κ system. The sample’s initial position is defined in motor coordinate space by 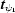. The sample is rotated about 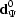, where 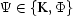, by 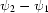 (1). Subsequent translation to the motor position coordinates 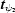 restores the sample to the centre without further changing its orientation (2).

Now assume a point-like sample at location vector 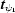 being centred at the 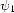 setting of one rotation axis while the other rotation-axis positions are zero valued. The transformation to the new location vector 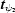 corresponds with rotation to the new angular position 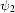. This may be calculated assuming perfect rotation in three-dimensional Cartesian space (Fig. 3 ▶) as 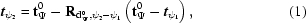
where 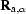 is the matrix representation of the rotation about the unit-length vector 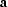 by the angle 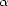. Note that the location vector 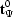 of the rotation axis is given by the translation motor positions when the rotation axis 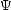 held by the translation stage is crossing the centring reference position, that is, at the intersection of the ω axis and the beam.

The translation correction 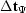 necessary to maintain centring while rotating about 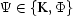 can thus be calculated as
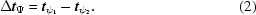
In a κ system, the κ arm holds the ϕ axis, and the direction and location vectors of the ϕ axis therefore depend on the angular position of the κ axis. Knowledge of the direction and location vectors at κ = 0° and ϕ = 0° thus permits the calculation of the new translation vector even after a complex movement combining multiple rotations about both the κ and ϕ axes [from 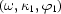 to 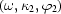]. The new translation vector can be expressed by following the sequence of rotating back the κ axis to 0°, applying the ϕ rotation and finally rotating κ to its new position as
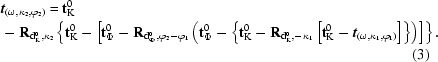

            

### 2.2. Translation calibration

This translation correction calculation requires accurate information about the direction vectors and locations of the rotation axes. These can be calculated for each axis by recording a set of translation points: 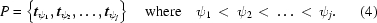
The ideal is a circular path as a point-like sample is rotated about the axis in question. Information about the accuracy of the system can be derived through statistical analysis of these points.

The assumptions made about the inherent error of the system define the approach used to model it. Here, three general categories describe potential sources of such errors.

(1) Anisotropy of the centring stage is one major source of non-ideality that encompasses problems related to relative movements between translation axes. Note that even in the case of anisotropy, the centring-stage motions are assumed to be linear. This assumption generally holds in practice.

(1*a*) Anisotropic scaling relates to the degree to which movements of the translation stage are interpreted by the system as real-space displacements. A system with scaling issues will produce real-space displacements of differing magnitudes along each axis even though all translation motors were instructed to move an equivalent amount.

(1*b*) Another source of anisotropy relates to non-orthogonality in the coordinate system. If the translation-stage axes do not move at right angles relative to one another, a variety of geometric problems will arise.

(2) The second category relates to the alignment of the coordinate systems in which translation and rotation movements are measured. The rotation-axis direction vectors can be determined in the space of isotropic translation-stage axes as well as in the space of a diffraction experiment by a rotation calibration (Paciorek *et al.*, 1999 ▶). If the orientations of the translation axes are properly aligned in the coordinate system of the rotation calibration, the two should match.

(3) The third category addresses both the accuracy with which rotation and translation are performed and the limitations of the visualization system, and thus directly relates to the accuracy and precision with which a centring is performed. Such measurement error directly affects the outcome of calibration by defining the precision of the system and is the most common source of error.

#### 2.2.1. Specific cases

The requisite size of the set of measured points 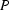 thus depends upon the theoretical assumptions made about the system. If the system is assumed to be isotropic and aligned with perfect rotation and centring measurements, only two unique points at two different angular settings must be measured. Since the direction vector 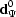 normal to the rotation plane can be known *a priori* from rotation calibration (Paciorek *et al.*, 1999 ▶), only the location of the rotation axis 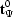 that is the centre of the circular path of the point needs to be determined. In this ideal case, the circle with a known central angle can be easily determined. Its centre can be calculated as follows: 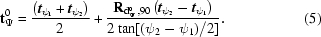
Also note that in the special case of rotating 180° between the two measurements, the degenerate solution results in the midpoint of the chord: 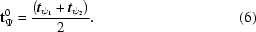
If the alignment of the centring stage itself is no longer considered perfect and the direction vector is unknown as a result, three measurements must be made in order to reconstruct the circle in three-dimensional space assuming that no other measurement error is present. The unit-length direction vector can be calculated as follows: 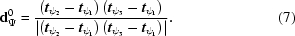
The same formulae (5) and (6) can be used to calculate the location of the axis 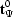, as for the previous case.

If the rotation is not free from angular errors, and so the angle 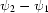 is not accurate, the three measurements forming a triangle are still enough for the calculation of the location of the axis by computing the circumcentre as the intersections of the perpendicular bisectors of the triangle.

#### 2.2.2. Generic case

In the presence of other measurement errors (*e.g.* when centring is no longer accurate), the point continues to trace a circular path due to the isotropy in the positioning stage. But additional points must be recorded to accurately fit a circle with such an inaccurate data set in three dimensions. If the random measurement error is sufficiently small relative to the radius of the circle, it can be averaged out by considering more points during circle fitting, as discussed below.

At least six points are required in the case of an anisotropic centring stage with accurate centring. Additional points must be collected in the case of an anisotropic centring stage with some error in the centring measurements.

(*a*) *Plane fit*. Ideally, each point falls on the same rotation plane **Π**, but this assumption cannot be made in reality. As such, **Π** is fitted to the points in the set 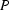 using principal component analysis (PCA) (Pearson, 1901 ▶), as implemented in *MATLAB* (MathWorks, 2009 ▶; Jolliffe, 2002 ▶). An alternative implementation involves the direct application of singular-value decomposition (SVD) (Golub & Reinisch, 1971 ▶) to the data for plane fitting. The PCA method returns the principal component coefficients or loadings required to define a plane in three dimensions. The first two principal component coefficients represent the basis vectors ***v*** and ***w*** that lie in **Π**, while the third principal component ***n*** is the normal vector to the plane. As the direction vector of the rotation axis is also normal to the plane, it is directly served as 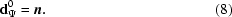
Since the mean of the data 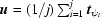 lies in **Π**, the location of the rotation axis 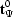 can be determined after a circle fit applied in **Π**, as its circumcentre. For this task, the point set 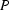 can be orthogonally projected 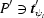 onto **Π** by using the coordinate transformation to the bases 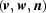 given by PCA and then projecting along the third coordinate: 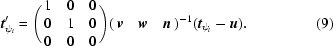

               

(*b*) *Ellipse fit*. Anisotropic scaling in translation space is always present to some degree and is particularly evident in the case of a poorly configured translation stage owing to the ellipse-shaped path traced by the sample upon rotation. Detecting such anisotropy is an excellent way to check for issues with the sample-positioning translation motors, and an ellipse fit is thus the most useful way to model the system. The semi-major and semi-minor axes calculated from the ellipse fit provide information regarding scaling by virtue of their relative magnitudes. Performing such a fit on a well configured system will generate axes of nearly equal magnitude, while a system with a scaling problem produces axes with very different magnitudes. Note that non-orthogonality of the translation system also results in an elliptical rotation path. The way of distinguishing between the two sources is discussed below.

In order to minimize the likelihood of an incorrect fit in the case of an automated calibration, a geometric fit of an ellipse in a parametric form is performed (Gander *et al.*, 1994 ▶). This algorithm uses the Gauss–Newton nonlinear least-squares approach to minimize the sum of the squares of the distances of the points in 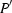 to an iteratively determined ellipse fit. This yields the parametric ellipse 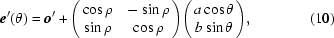
where 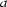 and 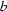 are the magnitudes of the semi-major and semi-minor axes, respectively, 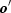 is the centre of the ellipse, 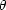 is the independent parametric angle and 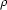 is the tilt angle of the ellipse in the plane about the centre point.

By applying the inverse of the transformation [equation (9)], the location of the approaching rotation axis can be calculated by converting the two-dimensional centre point coordinates 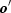 in **Π** back to the original three-dimensional translation space: 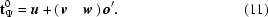

               

(*c*) *Scale-factor estimation*. The relative values of 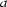 and 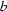 reveal a great deal about the state of the translation system. A successful plane and ellipse fit allow for the calculation of the anisotropic scaling factor for the coordinate system of the translation stage 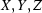. In order to calculate the anisotropy in the 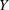 and 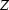 axes relative to the 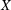 axis, the three-dimensional scale matrix 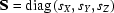 must be determined for 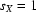, assuming that axis 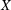 is correctly configured. The anisotropy-caused elliptical path of the point in the original translation space can be traced by mapping the set of ellipse points 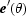 for 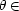 [0°, 360°] back to three-dimensional space, while keeping the centre point in the origin: 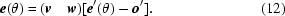
After applying the scaling correction, the data points lie on a circle, with a constant radius 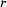. Hence, a series of equations can be set
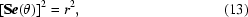
and solved using a linear least-squares approach. It provides the unknown diagonal elements of 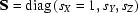 that describes the scaling of the 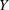 and 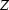 axes.

**Figure 4:**
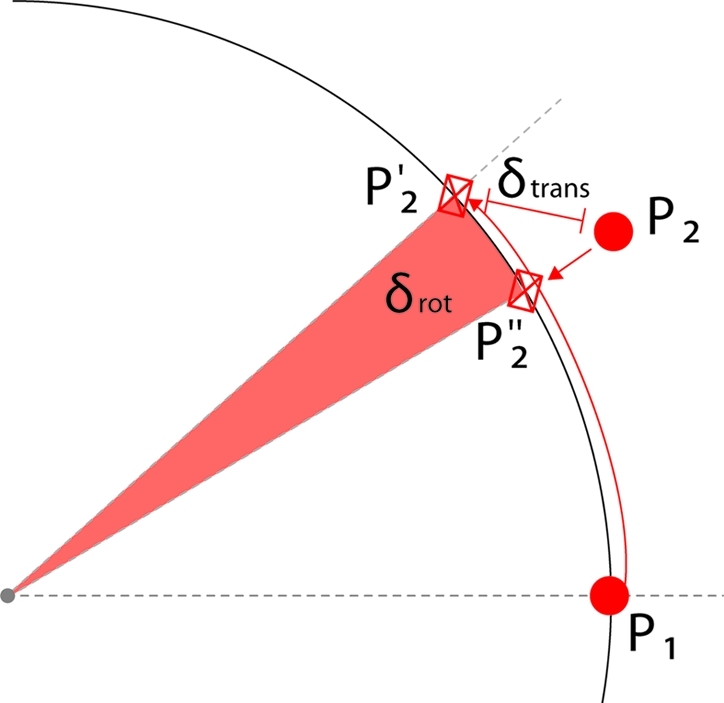
Calculation of linear and angular reconstruction errors. 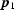 is the scale-corrected location of a measured point at the angular setting 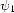. 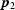 is similarly at the angular setting 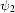. Assuming 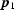 is correctly measured at 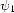, the expected location at 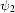 is shown as 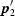. The three-dimensional distance between the expected (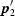) and measured (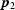) locations supplies the linear error δ_trans_. The projection of the measured point 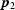 to the circle in the regression plane is shown as 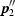. The angular difference between the expected and measured rotation provides the angular error (δ_rot_) of the measurement. Sample values of δ_rot_ and δ_trans_ are listed in Table 1 ▶.

However, non-negative constraints must be applied, as a negative scale factor is geometrically prohibited when assuming a right-handed orthogonal translation space. A standard error of regression higher than the expected measurement errors can highlight the presence of non-orthogonality in the system. The misalignment of the axes can be addressed by solving the same set of equations (13), but this time replacing the unknown scale matrix by an unknown base-transformation matrix.

(*d*) *Measure of error*. The scale factors are dimensionless and only provide relative information on the state of the system. If the observed ellipse is scaled to a circle by the appropriate scale factors, a linear distance error can be calculated. First, assume a nearly perfect position after scaling of some originally measured point in 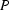. Let this point be 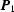 at the angular setting 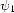. Corresponding to a new setting 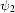, rotate this point about the calculated axis to its expected new location 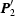. Let the position after scaling the measured point in 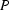 at 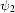 be 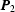. The three-dimensional distance between the expected 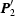 and measured 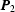 locations defines the linear distance error 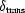 (Fig. 4 ▶). These errors represent real distances if the translation stage 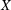 is properly configured as assumed when defining scale factor 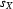 as unity. Hence, the linear distance errors indicate the expected accuracy of the translation correction.

Now, let the orthogonal projection of the point 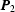 on a circle in the regression plane be 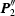. The angular difference between 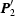 and 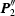 defines the angular error of that measurement 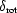 (Fig. 4 ▶). Systematic analysis of these measurement errors can point out specific problems, like issues related to backlash, slipping or improper scaling configurations as discussed below.

## 3. Discussion

The calibration method described in theory has been implemented in the goniometer-controlling software *STAC* (STrategy for Aligned Crystals). In practice, the calibration described above takes little time to perform on the beamline. This is especially true when combined with *STAC*, which offers a manual, guided or automatic solution for goniometer calibration. Such calibration can be performed as follows:

(i) Initialize and home the goniometer axes such that (ω, κ, ϕ) = (0°, 0°, 0°), and align the ω axis.

(ii) Perform centring (for example, using the on-axis microscope of an MD2 to avoid parallax error) on a well defined reference point that is clearly recognizable at all varieties of angles. After centring, the translation motor positions are registered such that 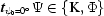.

(iii) Separately perform the following steps for the κ and ϕ axes, with the other set at 0°:

(*a*) Rotate about the given axis by an arbitrary angle *α*.

(*b*) Re-centre the reference point such that the translation position corresponding to the rotation is registered as 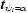.

(*c*) Repeat (*a*)/(*b*) at least once more if the direction vector is not known from *a priori* rotation calibration. In automatic mode, *STAC* repeats the procedure five more times, recording a total of six unique points evenly distributed and paired with another point 180° away.

(iv) If scale factors for a given axis are not equal to unity, adjust hardware or compensate for scaling in control software to remove anisotropy from the system configuration.

**Figure 5:**
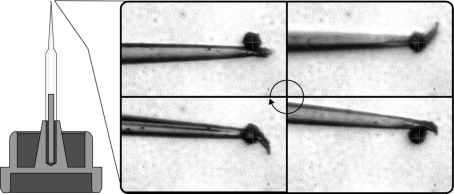
Schematic of a glass pin with bead. Images show a 10 µm polystyrene bead on a glass micropipette rotating 360° about the ϕ-axis post-centring. The bead has a greater diameter than the glass needle and allows for a clear visualization at all angles, thus ensuring precise centring.

The accuracy of the calculations derived from the TC procedure above is completely dependent on the ability of the operator to perform the requisite centring steps in a consistent fashion that minimizes measurement error. A special pin based on the SPINE standard (Cipriani *et al.*, 2006 ▶) has been designed in order to improve the centring accuracy and precision by overcoming problems related to the visualization of the point to be centred as it rotates out of the narrow focal plane at high magnification (Fig. 5 ▶). One may construct such a pin for TC by gluing a 10 µm-diameter polystyrene bead (Microbeads AS, Norway) to the tip of a borosilicate glass needle mounted on a crystal support. The capillary tube used to make the needle must have an inner diameter of around 0.8 mm to properly fit over the microtube on the support, and the needle must be pulled such that the outer diameter at the tip is around 5 µm. Conventional devices used to pull needles for *Xenopus* oocyte microinjections work well. Altering from the SPINE standard, one can glue the base of the capillary to the microtube at a slight angle. By leaving it shorter or longer than the specification, the path traced by the bead will be larger and thus easier to fit, dramatically improving the precision with which centring can be performed. Furthermore, with a large enough difference between the diameter of the needle tip and the diameter of the bead, centring will be possible even when the κ arm is set to a large angle where a typical sample would be obscured. This is a critical shortcoming of previous standards used for calibration, such as capillary tubes or acupuncture needles affixed to a sample support base. Both types of pins often feature excellent points for visual centring, but opening the κ arm results in their obstruction by the rest of the system. The precision with which manual centring is performed is also improved by incorporating a circular-shaped reticule into the centring software which is scaled to the on-screen size of the bead. This allows for precise identification of the bead centre at all orientations and even at locations outside the focal plane of the visualization system. Automated centring is possible using edge-detection or circle-shape recognition. An algorithm to perform reliable centring has already been implemented in the crystal centring software *C3D* (Lavault *et al.*, 2006 ▶).

**Figure 6:**
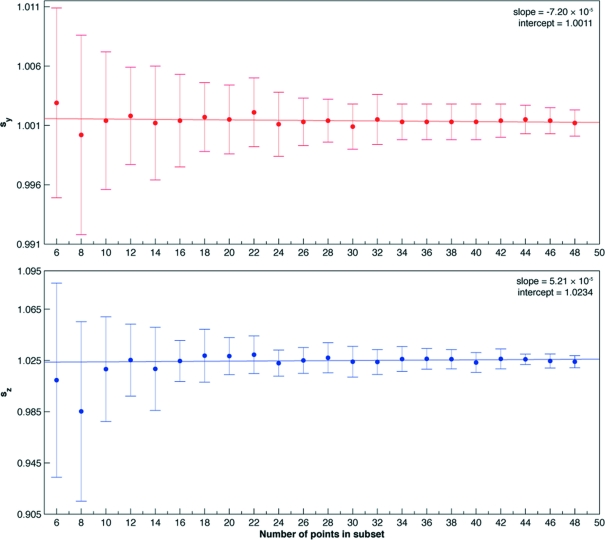
Error in calculation of scale factors *s_Y_* and *s_Z_* for rotation about the κ axis as a function of the number of data used for the ellipse-fit calculation. Points were recorded at 5° intervals over [0°, 240°] for a total of 49 points. Scale factors were calculated for subsets of these points; for a six-point subset, three points were chosen randomly from evenly distributed ranges on the rotational path, and the following three were points located exactly 180° away. If a randomly selected point had no pair (*i.e.* any point on the interval [60°, 180°]), another point with no pair was selected as well. This random selection and scale-factor calculation was repeated 100 times for each subset. The means of resulting scale factors are plotted together with the standard deviations as a function of the number of points in the subsets.

One aspect of the TC method presented here that illustrates its versatility is the speed with which the calibration can be performed. Using automated centring, the calibration can be performed automatically in less than 15 min. Although increasing the number of points sampled around the rotational path of the sample increases the accuracy of the subsequent calculations, only three evenly distributed pairs of points separated from one another by 180° must be collected for rotations about any rotation axis for the ellipse-fit algorithm to work in a robust manner (Fig. 6 ▶). The accuracy of the ellipse fit is evaluated based on the stability with which the scale factors are provided. Collecting an additional three pairs for a total of 12 points is recommended for more precise calibration, as the influence of outlying points is decreased substantially. Outlier detection and elimination algorithms like the confidence coefficient assignment or inward procedure summarized by Ben-Gal (2005 ▶) could reduce the number of data points needed. Calibration data for the κ axis should be collected in a similar fashion, although pairs may not be available for points over a certain interval because of possible collisions or instrument limitations. For the EMBL/ESRF MK3, points in the interval [80°, 180°] cannot have pairs, because the κ arm is limited to a maximum of 260°.

An implementation of the method described above can be used to rapidly process TC data and produces statistics similar to those presented (Table 1 ▶).

**Figure 7:**
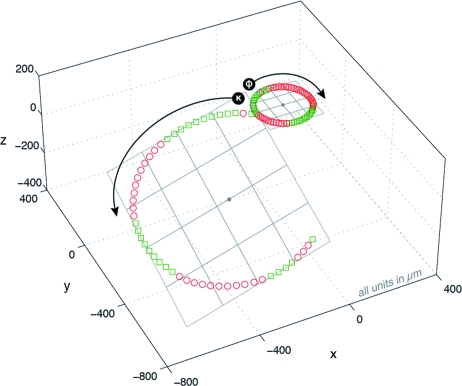
Paths traced by κ and ϕ axes during rotation and accompanying plane fits. The ϕ path lies in the *XY* plane, while the κ path traces a plane in all three dimensions. Points denoted by green squares fall below the best-fit plane, while points denoted by red circles lie above the best-fit plane. Statistics for these plane fits are listed in Table 1 ▶(*a*).

Data were measured using an EMBL/ESRF mini-κ goniometer head (MK3) installed on an MD2M diffractometer on ID14-4 at ESRF (McCarthy *et al.*, 2009 ▶). Motor positions were recorded every 5° following the re-centring of a mounted calibration bead (see Fig. 5 ▶) while rotating about the ϕ axis over the range [0°, 360°] followed by the κ axis over the range [0°, 240°]. The measured data are shown in Fig. 7 ▶. The data for presenting statistics on a misconfigured system (Table 1 ▶ *b*) were produced by artificially introducing 10:7 anisotropy in the *Y*-motor configuration. Note that the identical motors *X* and *Y* are from the two-dimensional centring stage, while the motor *Z* translating along the ω axis is part of the three-dimensional alignment carriage.

The plane fit shows submicron deviations of the data from an ideal planar rotation in all cases. The error is even less pronounced in the case of the ϕ axis, as it is mechanically less strained than the κ axis and mainly rotates in the *XY* plane (see Fig. 7 ▶). Hence, the errors contributed from the independent *Z* motor are insignificant. The normal vectors of the best-fit planes are (−0.0094, 0.0045, −0.9999) and (−0.2903, −0.2887, 0.9123) for ϕ and κ, respectively. The angle between these normal vectors should always be around 24° in the case of the MK3. Deviation from such nominal value (Fig. 2 ▶) can indicate the presence of a scaling problem in the translation motor configurations. Note that MK3 is designed as a re-mountable goniometer head, and as such does not have additional nominal parameters for the location and orientation of axes. In contrast, comparison to previous calibration results can help in identifying potential problems.

Before the ellipse fit, data are orthogonally projected onto the fit plane and converted to a set of two-dimensional points. The origin of the coordinate system in the plane is chosen to be at the mean of the data. By measuring pairs 180° apart, the mean of the data should fall close to the centre of the ellipse fit. Large distances of almost 400 µm in the case of the κ axis result from the fact that the data points were not collected in such pairs (see Fig. 7 ▶). While the difference between the radii of the ellipse fit in Table 1 ▶(*b*) suggests a potential misconfiguration of the motors, data in Table 1 ▶(*a*) illustrate a more balanced system.

Projecting the ellipse back to the original three-dimensional space, scale factors are calculated to stretch the ellipse onto a circular path. Scale-factor calculation reveals the strong anisotropy introduced into the system in Table 1 ▶(*b*). Note that even a small inherent anisotropy is precisely computed for the *Z* axis using the data about κ. The calculated 2.4% scale correction along the *Z* axis shows a slight error in the configuration of the *Z* motor belonging to the alignment carriage (see Fig. 1 ▶ *b*), but differing from the *X* and *Y* motors of the centring stage. Also note that data collected about the ϕ axis do not provide any information regarding the *Z* axis. As such, the calculation does not provide a solution in this degenerate case. After correcting the data for misconfigured scaling, a simple geometric circle-fit algorithm identifies the correct circular path in both isotropic and anisotropic cases.

The calculated angular and linear distance errors are also realistic and consistent with one another in both the isotropic and anisotropic cases. The angular reproducibility of the MK3 motors has been measured as 0.1° for κ and 0.04° for ϕ, while the diameter of the maximum sphere of confusion (SOC) for the whole goniometer including the MK3 head was determined to be less than 6 µm. Opening the κ arm results in a larger SOC owing to the increased mechanical strain on the MK (Fig. 2 ▶). The distribution of the linear distance errors is an indication of the precision expected from a translation correction derived from a TC. Here, their magnitude, as presented in Table 1 ▶, is close to the diameter of the SOC. In such a setup with precision mechanics, the primary source of error is due to the centring governed by the SOC. A vertical goniometer setup resolves this issue, and the SOC of the same MK3 can be reduced to submicron levels, as demonstrated on the ID29 and ID23-2 beamlines at ESRF (data not shown).

Although goniometer systems such as the MK3 are quite robust and undergo very little drift over weeks of activity, the ease with which TC can be performed makes it an excellent way to detect occasional instrumentation problems that arise in hardware or software. The biggest source of such errors relates not to protracted data collection and general wear and tear, but rather to the different incremental changes made intentionally and unintentionally to the sample-positioning system by users and support staff. Updates to device control software could also potentially reset previously established configurations.

The TC procedure yields valuable information which can be used to address many of these problems. First, the plane fit can be used to visualize the ideality of sample rotation as it should always move in a plane. Furthermore, the calculated scale factors may be used to detect anisotropy in the reference frame caused by the sample-positioning motors. Note that in the case of the ϕ axis the direction vector of the rotation is normally set orthogonal to the two-dimensional centring-stage axes. Incompatible scaling configuration of the two motors can be directly read out from the ellipse-fit radii. This simple relationship does not hold in the case of the third axis, for which a separate step of scale-factor calculation must be performed from data around κ.

The potential problem of a non-orthogonal centring stage can also arise, especially in systems where the translation stages are not directly coupled. The combined use of the re-purposed alignment carriage and a two-dimensional centring table is such a case. Although the least-squares error determined during a scale-factor calculation can be indicative of such a situation, we have not experienced a problem like this on any of the ESRF MX beamlines equipped with MD2 or MD2M diffractometers.

In most cases, the system should be sufficiently isotropic with proper scaling performed by the software, yielding a sample path that is circular. Once a circle fit has been performed, additional statistics about the linearity of the system can be analysed. The linear reconstruction error for a given axis reveals the accuracy with which the point can be automatically re-centred in microns, while the angular uncertainty relates to the accuracy with which the rotation is being performed. These metrics, when compared with the expectations for a given hardware setup, can highlight underlying problems, like losing steps, or having a mechanical flaw which may even vary slightly under different physical constraints.

## 4. Conclusion

This study provides a simple and robust method for the calibration of an inverse-κ goniometer sitting on a translational positioning system responsible for re-centring a sample. Following the collection of only a few data, the method described here can be used to assess and hone the precision of the given goniometer setup. Beamline-control software solutions like *STAC*, that implement the calibration procedure, can be used in combination with automated centring software like *C3D*. As a result, TC with a calibration pin and a standard microscopy-based sample-centring system can be fully automated. In the case of the EMBL/ESRF-designed mini-κ goniometer, this TC method results in a system that is accurate to the level needed to perform even the most orientation-sensitive experiments in MX. Indeed, the calibration is simply limited by the accuracy with which the centring is performed with a calibration pin and is thus far more accurate than the centring performed on a typical sample. Taking advantage of the calibration results, sample centring can be automatically maintained across all reorientations (as implemented by *STAC*). Furthermore, integrating an automatic translation-correction procedure with an experiment control system like *MxCuBE* (Macromolecular Crystallography Customized Beamline Environment) (Gabadinho *et al.*, 2010 ▶) and the online data analysis system *EDNA* (Incardona *et al.*, 2009 ▶) can hide the reorientation complexity of inverse-κ systems, and allow their use as pure rotational goniometers.<!?tpb=-12pt>

## Supplementary Material

Stereogram for three-dimensional visualization of Fig. 2. DOI: 10.1107/S0108767311004831/zm5081sup1.tif

## Figures and Tables

**Table 1:** Statistics returned from processing TC data collected with the mini-κ (MK3) on ID14-4 at ESRF The root-mean-square deviation (RMSD) of the plane fit together with the maximum absolute deviation (MAX) shows the goodness of fit. From the ellipse fit of the planar data, the distance (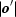) between the mean of the data (the origin on the two-dimensional plane) and the centre of the ellipse (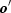), as well as the lengths of the semi-major and semi-minor axes *a* and *b* are presented. The scale factors *s_XYZ_* along each ordinate calculated from the ellipse fit relate the appropriate scaling needed to transform the rotation path into a circle. The radius *r* is provided by a circle fit for the scaled data. RMSD and MAX of the linear distance errors δ_trans_ and angular errors δ_rot_ are shown to describe their distribution and relate the overall calibration state of the system. The values in (*a*) suggest a well calibrated system. The ellipse fit yields a nearly circular path, and very little scaling is required to transform the data. Errors are within the limitations of the system. The data in (*b*) were produced by artificially introducing 10:7 anisotropy in the *Y* axis relative to the *X* axis to simulate an error in sample-positioning motor configuration.

Axis	Plane fit	Ellipse fit (two-dimensional)			Scale factors	Scaled circle fit	Scaled errors	
	RMSD (MAX) (µm)	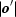 (µm)	*a* (µm)	*b* (µm)	*s_XYZ_*	*r* (µm)	δ_trans_ RMSD (MAX) (µm)	δ_rot_ RMSD (MAX) (°)
(*a*)								
κ	0.85 (1.86)	398.9	1025.7	1021.6	(1 1.0007 1.0239)	1026.1	9.06 (14.19)	0.48 (0.78)
ϕ	0.66 (1.75)	19.5	674.7	669.8	(1 1.0074 —)	675.0	3.57 (6.58)	0.26 (0.54)
								
(*b*)								
κ	0.84 (1.84)	380.5	1024.0	747.4	(1 1.4299 1.0237)	1026.2	9.03 (14.19)	0.47 (0.78)
ϕ	0.65 (1.73)	17.3	674.6	468.9	(1 1.4387 —)	674.9	3.57 (6.65)	0.26 (0.54)

## Footnotes

1 A supplementary stereogram image for three-dimensional visualization is available from the IUCr electronic archives (Reference ZM5081). Services for accessing these archives are described at the back of the journal.
